# Expression of the bile acid receptor FXR in Barrett's esophagus and enhancement of apoptosis by guggulsterone in vitro

**DOI:** 10.1186/1476-4598-5-48

**Published:** 2006-10-20

**Authors:** Andrea De Gottardi, Jean-Marc Dumonceau, Fabien Bruttin, Alain Vonlaufen, Isabelle Morard, Laurent Spahr, Laura Rubbia-Brandt, Jean-Louis Frossard, Winand NM Dinjens, Peter S Rabinovitch, Antoine Hadengue

**Affiliations:** 1 Division of Gastroenterology, University Hospital, Micheli-du-Crest 24, 1205 Geneva, Switzerland; 2 Division of Clinical Pathology, University Medical Centre, Michel Servet 1, 1211 Geneva, Switzerland; 3 Department of Pathology, Josephine Nefkens Institute, Erasmus Medical Center, P.O. Box 1738, 3000 DR Rotterdam, The Netherlands; 4 Department of Pathology, 1959 NE Pacific Avenue HSB K-081, Box 357470, Seattle, Washington 98195-7705 USA

## Abstract

**Background:**

Barrett's esophagus, a risk factor for esophageal adenocarcinoma, is associated with reflux disease. The aim of this study was to assess the expression of bile acid receptors in the esophagus (normal, esophagitis, Barrett's esophagus and adenocarcinoma) and to investigate their possible function.

**Results:**

the expression of the bile acid receptors FXR and VDR in esophageal biopsies from patients with a normal mucosa, esophagitis, Barrett's esophagus or adenocarcinoma (n = 6 per group) and in cell lines derived from Barrett's esophagus and esophageal adenocarcinoma, was assessed by real time Q-PCR and immunohistochemistry. The effect of guggulsterone, an antagonist of bile acid receptors, on apoptosis of Barrett's esophagus-derived cells was assessed morphologically, by flow cytometry and by measuring caspase 3 activity.

The expression of FXR was increased in esophagitis, Barrett's esophagus and adenocarcinoma compared to normal mucosa by a mean of 44, 84 and 16, respectively. Immunohistochemistry showed a weak expression in normal esophagus, a strong focal reactivity in Barrett's esophagus, and was negative in adenocarcinoma. VDR expression did not significantly differ between groups. In cell cultures, the expression of FXR was high in Barrett's esophagus-derived cells and almost undetectable in adenocarcinoma-derived cells, whereas VDR expression in these cell lines was not significantly different. *In vitro *treatment with guggulsterone was associated with a significant increase in the percentage of apoptotic cells and of the caspase 3 activity.

**Conclusion:**

the bile acid receptor FXR is significantly overexpressed in Barrett's esophagus compared to normal mucosa, esophagitis and esophageal adenocarcinoma. The induction of apoptosis by guggulsterone in a Barrett's esophagus-derived cell line suggests that FXR may contribute to the regulation of apoptosis.

## Background

Barrett's esophagus (BE), defined by the detection of intestinal metaplasia in the esophagus at histological examination [1], is the most important risk factor for esophageal adenocarcinoma (AC) [2]. In subjects with BE the annual incidence of esophageal AC is approximately 0.4–2.1% [3]. Prevailing theories link this type of cancer to gastroesophageal reflux disease (GERD). The noxious agents responsible for injuring the esophageal mucosa in patients with GERD may originate from the stomach (hydrochloric acid and pepsin) or the duodenum (bile acids and pancreatic secretions). Mixed bile and acid reflux is more harmful to the esophageal mucosa than acid reflux alone in humans [4,5]. In an experimental rat model, the creation of a duodenoesophageal anastomosis led to esophagitis, intestinal metaplasia and eventually esophageal AC [6], and biliary diversion did not lead to regression of BE, but prevented AC [7]. Previous studies have demonstrated that the transformation of BE into esophageal AC is related to a loss of apoptotic mechanisms [8]. However, the contribution of bile acid receptors to the apoptotic processes in BE and esophageal AC is unknown.

Bile acid receptors, including the farnesoid X receptor (FXR) and the vitamin D receptor (VDR), have recently been identified [9,10]. These transcription factors are abundantly expressed in the liver and in the lower digestive tract, where they regulate the homeostasis of cholesterol and bile acids [11]. In the human colon, the expression of FXR progressively decreases in the sequence normal mucosa – adenoma – adenocarcinoma [12].

The observation that apoptosis may be triggered by guggulsterone, a known FXR antagonist [13], suggests that this compound may enhance apoptosis in tissues and cells expressing this receptor. VDR is best known for maintenance of mineral homeostasis and bone architecture, but its role as a mediator of apoptosis has been increasingly recognised [14] in various types of cells. The expression and the possible function of bile acid receptors in the esophagus and in particular in BE and esophageal AC is unknown.

In this study we hypothesized that FXR and VDR are expressed in the esophagus and may contribute to the regulation of apoptosis in intestinal metaplasia. To test this hypothesis, we measured the expression of these receptors by quantitative polymerase chain reaction (PCR) and immunohistochemistry in esophageal samples from patients with a normal esophagus, esophagitis, BE or AC, as well as in cell lines derived from human BE and from esophagus AC. Finally, we investigated *in vitro *a possible role of esophageal bile acid receptors on apoptosis, using FXR and VDR agonists and antagonists.

## Results

### Expression of FXR and VDR in tissue biopsies

**Figure 1:**
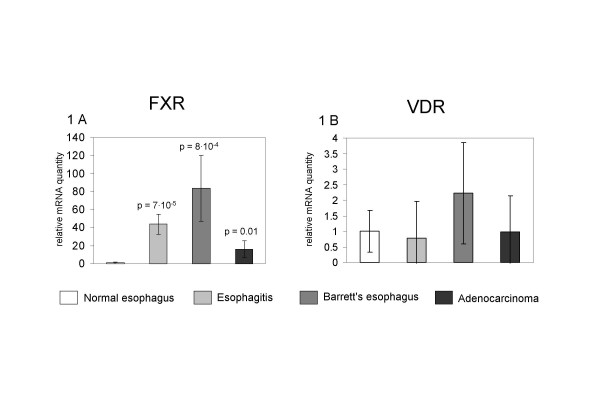
Relative expression of mRNA coding for FXR (1A) and VDR (1B) in esophageal biopsies from normal esophagus, esophagitis, Barrett's esophagus and esophageal adenocarcinoma, n = 6 for each group.

There was an increase in the relative expression of FXR in the sequence from normal esophagus (1 ± 0.83) to esophagitis (44 ± 15, p = 7·10^-5^) and to BE in which this receptor resulted most elevated (84 ± 36, p = 8·10^-4^). In AC, the expression of FXR was inferior to that measured in esophagitis and BE, but it was significantly superior to that of normal esophagus (16 ± 9, p = 0.01) (Figure 1A).

The relative quantity of VDR mRNA was not significantly different in normal esophagus (1 ± 0.67) compared to esophagitis (0.78 ± 0.89, p = ns), BE (2.23 ± 1.62, p = ns) and AC (0.97 ± 0.99, p = ns), suggesting a constitutive expression of this receptor in these different esophageal conditions (Figure 1B).

### Expression of FXR and VDR in BE-derived and esophageal AC-derived cell lines

**Figure 2:**
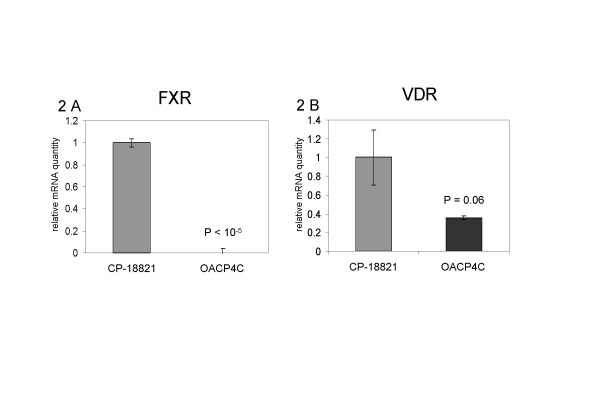
Relative expression of mRNA coding for FXR (2A) and VDR (2B) in cultures of cells derived from Barrett's esophagus (CP-18821) and esophageal adenocarcinoma (OACP4C).

The expression of FXR was significantly higher in BE-derived cells compared to AC-derived cells (1 ± 0.05 vs 9·10^-4 ^± 1·10^-4^, p = 3·10^-6^) (Figure 2A). This difference suggests an almost complete loss of expression of FXR in esophageal AC cells.

VDR expression was inferior in AC compared to BE cells, although this difference did not reach statistical significance (0.36 ± 0.02 vs 1 ± 0.28, p = 0.06), (Figure 2B).

### Localisation of FXR by immunohistochemistry

**Figure 3:**
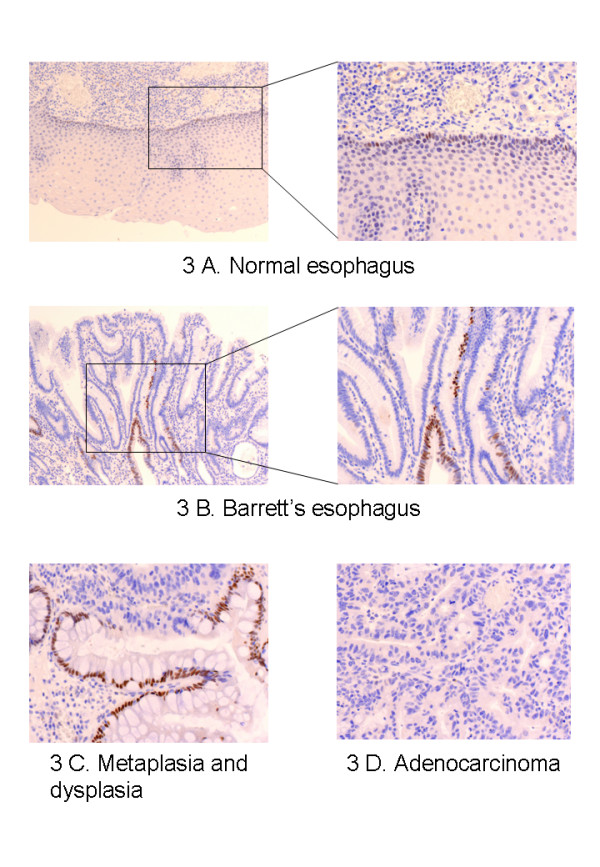
Representative immunohistochemistry images using a monoclonal FXR antibody on tissues from a normal esophagus (3A), Barrett's esophagus (3B), Barrett's esophagus and dysplasia (3C) and esophageal adenocarcinoma (3D). Weakly stained nuclei were present in the basal layer of squamous epithelium in normal esophagus and a strong staining was observed in focal areas of Barrett's esophagus. Dysplasia and adenocarcinoma remained negative.

In tissue samples of normal esophagus (Figure 3A) there was a weak positive signal of the majority of cellular nuclei, which was limited to the basal layer of the squamous epithelium. The superficial epithelial layers and the lamina propria were characterized by a negative staining. In Barrett's esophagus (Figure 3B), there was a strongly positive nuclear staining in some areas, while other metaplastic regions remained negative. The focally positive areas were apparently randomly distributed in the crypts and the positive cells were morphologically indistinguishable from those which did not stain. Tissue from dysplasia (Figure 3C) or esophageal adenocarcinoma (Figure 3D) resulted completely negative.

### Induction of apoptosis

**Figure 4:**
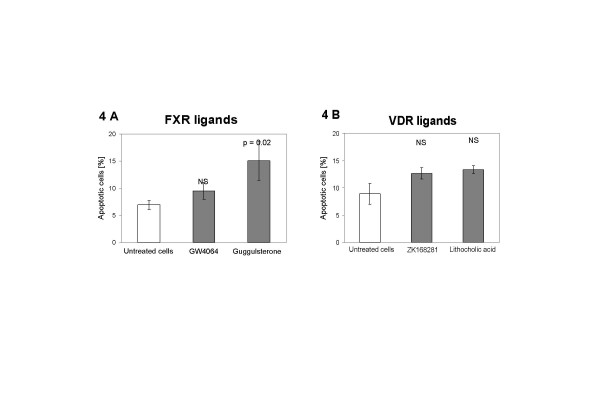
Percentages of apoptotic CP-18821 Barrett's esophagus-derived cells after treatment with FXR (4A) or VDR (4B) ligands, as measured by flow cytometry.

**Figure 5:**
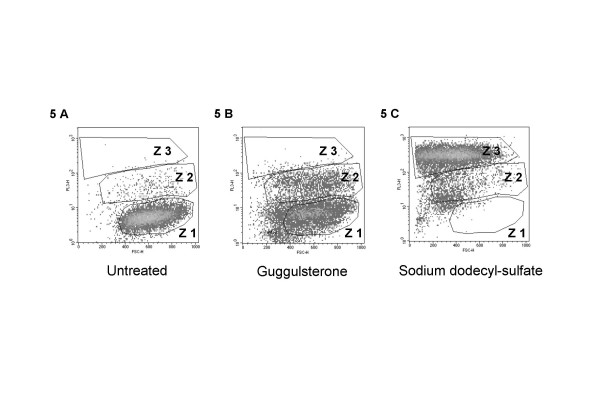
Representative FACS diagrams of cells derived from Barrett's esophagus (CP-18821): without treatment (5A), treated with guggulsterone (5B) and treated with sodium dodecyl-sulfate-treated (5C). Zone 1 (Z 1): cells alive, Z 2: apoptotic cells. Z 3: necrotic cells.

Treatment of the BE-derived cells with the FXR agonist GW 4064 did not significantly affect the percentage of apoptotic cells compared to untreated cells (9.5% ± 1.6% vs. 6.9% ± 0.8%, p = ns; Figure 4A). On the other hand, treatment with the FXR antagonist guggulsterone resulted in an increased proportion of cells undergoing apoptosis (15.1% ± 3.5% vs 6.9% ± 0.8%, p = 0.02; Figure 4A), without any induction of necrosis, as assessed by flow cytometry (Figure 5).

VDR stimulation or inhibition by lithocholic acid or ZK 168281, respectively, did not significantly alter the proportion of apoptotic cells (Figure 4B).

**Figure 6:**
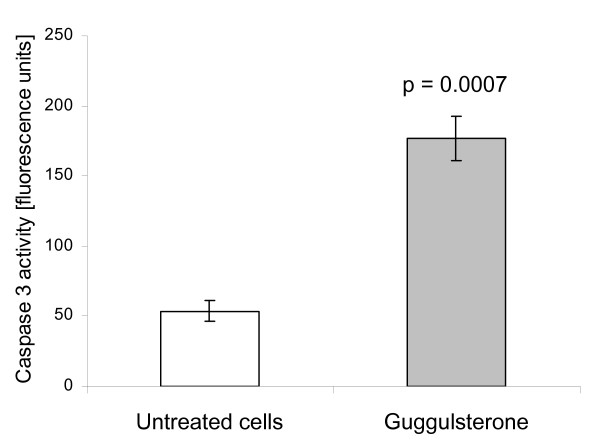
Fluorometric measurement of caspase 3 activity in CP 18821 Barrett's esophagus-derived cells was significantly increased after treatment with guggulsterone, compared to untreated cells.

The significant proapoptotic effect of guggulsterone on BE-derived cells (CP-18821) previously demonstrated by flow cytometry was additionally verified by measuring the caspase 3 activity. Guggulsterone treatment was associated with a significant induction of apoptosis compared to untreated cells (177 ± 15 vs 53 ± 7; p = 7·10^-4^) as measured fluorometrically (Figure 6).

**Figure 7:**
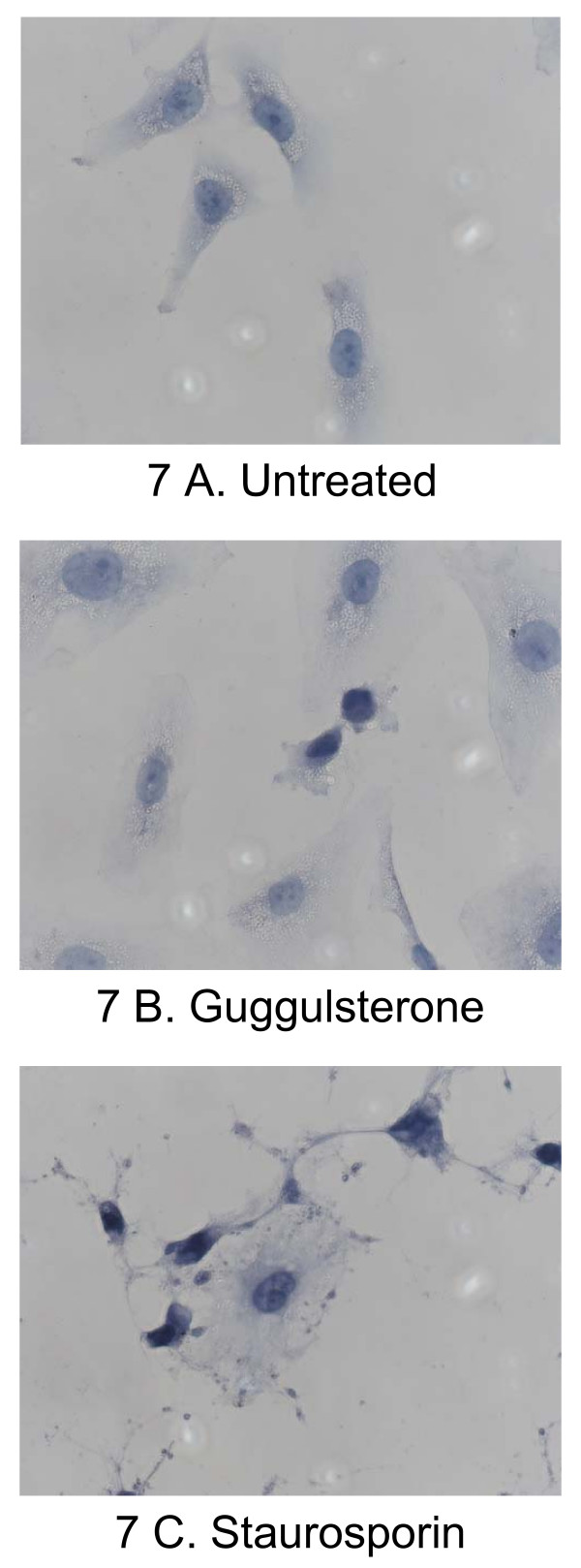
Representative microscopy photographs showing normal untreated Barrett's esophagus-derived cells (7A) and cell shrinkage and condensation of nuclei in cells incubated with guggulsterone (7B) or with staurosporin (7C), an inducer of apoptosis.

Finally, the induction of apoptosis was qualitatively assessed by observing the morphological changes after treatment of cells with guggulsterone (Figure 7). A considerable proportion of these cells showed shrinkage and nuclear condensation (Figure 7B). Staurosporin induced similar, but more pronounced changes (Figure 7C).

## Discussion

We showed that the bile acid receptor FXR is significantly more expressed in tissue biopsies from BE as compared to normal esophageal mucosa. This is probably related to the replacement of the normal squamous epithelium by intestinal metaplasia in BE (FXR is abundantly expressed in small and large intestine mucosa [15,16]), although the role of other factors (e.g. age, gender) cannot be excluded. Immunohistochemistry showed that FXR is weakly present and limited to the nuclei of the basal cell layer in normal esophagus. FXR was strongly present in BE, but its distribution was focal, suggesting the presence of clusters of cells in which this nuclear receptor was highly expressed and others where FXR was absent, although positive and negative cells were morphologically impossible to differentiate. Staining of tissues presenting dysplasia or carcinoma was in all cases negative. These findings suggest that FXR may be a heterogeneous marker for Barrett's metaplasia, but not dysplasia or adenocarcinoma. The increase of FXR expression in biopsies from patients with an esophagitis is an unexpected finding, because it is not a columnar epithelium. A possible explanation is that the presence of inflammation may enhance the expression of FXR. Esophagitis is a risk condition for the development of Barrett's esophagus: the increase in FXR expression may possibly occur prior to the metaplastic changes from a squamous into a columnar epithelium.

FXR is a nuclear receptor activated by bile acids, in particular chenodeoxycholic acid, which are abundantly present in bile, a component of refluxate in BE [5,6]. This receptor has been suggested to play a role in the regulation of apoptotic pathways [17], which are of particular relevance in BE. Indeed, in BE, cells present a decreased capability of apoptosis compared to cells from esophageal squamous epithelium. This is suggested to relate to an increased expression of antiapoptotic proteins such as Bcl-xl and Bcl-2, as well as a decrease in proapoptotic factors such as Bax [8,18,19]. Avoidance of apoptosis is an important mechanism in the multistep process which characterizes the progression of BE towards AC [20]. As a consequence, compounds capable of inducing apoptosis in BE could be beneficial in decreasing the risk of malignant degeneration.

Guggulsterone, a potent antagonist of FXR [21], significantly induced apoptosis in BE-derived cells. A similar induction of apoptosis by guggulsterone has been reported in human cells derived from lung carcinoma and leukaemia. This has been attributed to a down regulation of genes known to stimulate cell proliferation such as cyclin D1 and c-myc, as well as to a decrease in gene products involved in antiapoptotic pathways (Bcl-2, c-FLIP and survivin), suggesting that the stimulation of apoptosis might be an effect of guggulsterone-mediated FXR inhibition [13]. However, we cannot exclude that apoptosis in our experiments was induced by other mechanisms. In fact, guggulsterone, as a steroid receptor ligand [22], may also have induced apoptosis of BE-cells by acting on these receptors, but the distribution and regulation of steroid receptors in normal esophagus and BE is not known.

When treated with the FXR agonist GW4064 (which is a more potent agonist than chenodeoxycholic acid), BE-derived cells disclosed a slight, not significant increase of apoptosis. The use of FXR agonists as enhancers of apoptosis has been described in ovarian cancer cells [23] and in vascular smooth muscle cells [24]. Our results are in agreement with these findings, although the increase in apoptosis observed in our experiments, which was statistically not significant, may be due to a different sensitivity to GW4064 of the cell line we used.

In esophageal biopsies from AC, in the corresponding cell cultures (OACP 4C) and in surgical tissue samples, very low levels of expression of FXR, compared to BE, were detected by PCR and imunohistochemistry. This could be due to the low grade of differentiation observed in our AC tissue samples at histological examination, as well as to the marked pleiomorphism of cell cultures, which is possibly associated with a loss of expression of FXR. Interestingly, we previously observed a similar reduction in FXR expression in colon adenoma and adenocarcinoma, compared to normal colon mucosa [12], supporting the hypothesis that FXR is down-regulated in poorly differentiated tissues and cells of tumoral origin.

The expression of VDR was similar in the different groups of patients, and VDR agonists/antagonists were not found to significantly modify apoptosis. This suggests that VDR is constitutively expressed and is not a major player in the regulation of apoptosis in BE.

A limitation of this study is that cell cultures were continuously exposed to guggulsterone at a neutral pH. Under physiological conditions the human esophagus is exposed to bile reflux for intermittent periods of time (in the range of minutes) in association or not with acid reflux. Nehra et al. reported total bile acid concentrations of 180 μM and a percentage of time at pH<4 of 22% in the esophagus of patients with BE during a 15-hour monitoring study [4]. This drawback could be overcome by studying apoptotic mechanisms of BE in an animal model [25] treated with the compounds used in this study.

## Conclusion

In summary, the results of this study indicate that the bile acid receptor FXR is focally overexpressed in BE and that treatment with guggulsterone, an FXR antagonist, significantly enhances apoptosis in a human BE-derived cell line. We conclude that FXR inhibition may represent a novel therapeutic approach in BE and that this warrants *in vivo *studies.

## Methods

### Patients

Esophageal tissue biopsies were obtained during upper digestive endoscopy from 24 patients with a normal esophagus, reflux esophagitis, BE or AC (n = 6 for each group). Tissue samples were taken during endoscopy for histological examination and additional tissue samples were stored in RNAlater™ (Qiagen, Hombrechtikon, Switzerland) and frozen at -70°C. For each patient, the diagnosis was confirmed histologically. Biopsies of BE presented no or low-grade dysplasia and all AC were moderately to poorly differentiated. The research protocol has been approved by the Ethics Committee of Geneva University Hospital, and written informed consent was obtained from all patients.

Patient's characteristics including the Reflux Symptom Index Score [26] and intake of proton pump inhibitors during the 7 days preceding endoscopy are shown in Table 1.

**Table 1:** Patients' characteristics

	Normal esophagus (n = 6)	Reflux esophagitis (n = 6)	Barrett's esophagus (n = 6)	Esophagus adenocarcinoma (n = 6)
Age [years]	55 ± 7	50 ± 9	78 ± 3	79 ± 8
Male [%]	17	50	67	67
Reflux symptom index score	1.9 ± 0.9	3.8 ± 1.8	1.3 ± 1.2	1.5 ± 1
Proton pump inhibitor medication [%]	33	67	67	100
Smoker [%]	33	33	33	33
Alcohol intake >20 g/d [%]	17	67	50	33
Caucasian ethnicity [%]	100	100	100	100

### Cell cultures

Primary cell cultures from BE (CP-18821) transduced with a retrovirus containing the human catalytic subunit of telomerase reverse transcriptase [27] were maintained at 37°C in 5% CO_2 _atmosphere in MCDB 153 medium supplemented with 5% fetal bovine serum, 0.4 μg/mL hydrocortisone, 10^-10 ^mol/L cholera toxin, 140 μg/mL bovine pituitary extract, 20 μg/mL adenine, 5 μg/mL insulin, 5 μg/mL transferrin, 5 μg/mL selenium (all from Sigma, Buchs, Switzerland), 4 mmol/L glutamine, 20 ng/mL epidermal growth factor, 100 U/mL penicillin, 100 μg/mL streptomycin and 0.25 μg/mL amphotericin B (all from Gibco, Basel, Switzerland).

The human gastro-esophageal junction AC-derived cell line OACP 4C [28] and the epithelial cell line derived from human colorectal adenocarcinoma HT-29 (ECACC, Salisbury, UK) were maintained at 37°C in 5% CO_2 _atmosphere in RPMI-1640 (Sigma) medium supplemented with 10% fetal bovine serum, 100 U/mL penicillin and 100 μg/mL streptomycin (Gibco). HT-29 cells served as a positive control for the quantitative real time PCR [12].

### RNA isolation, reverse transcription and quantitative PCR

Total RNA was extracted from tissue biopsies weighing 5-10 mg using the RNeasy Mini Kit (Sigma) according to the manufacturer's instructions. cDNA was synthesized from 1 μg total RNA with the Omniscript reverse transcriptase (Qiagen), an RNAse inhibitor and random hexamers (Promega, Wallisellen, Switzerland). A LightCycler system (Roche, Basel, Switzerland) was used for real time PCR. The relative quantification of gene expression was normalized to the endogenous ribosomal 18S RNA. Amplification was performed in a total volume of 10 μL containing 5 μL of kit-supplied QuantiTect™ Master mix 2×, 0.5 μL of specific primer mix, 0.5 μL of QuantiProbe, 3 μL RNase-free water and 1 μL of cDNA sample.

The following primers were used for FXR gene amplification: F 5'-GGACCATGAAGACCAGATT-3' and R 5'-ATGCCCAGACGGAAGTTT-3', with a 5'-GCTATGTTCCTTCGTT-3' probe. The expression of VDR was measured using the following primers: F 5'-TGACCCTGGAGACTTTGA-3' and R 5'-TCGCCTGAAGAAGCCTTT-3', with a 5'-CTGTGGGGTGTGTGGA-3' probe. PCR amplification curves were analyzed with the LightCycler Software version 3.5.28.

### Immunohistochemistry

Resection specimens of 8 patients with Barrett's carcinoma, all containing normal esophagus, metaplasia, dysplasia and adenocarcinoma were stained. Four-micrometer paraffin sections were dewaxed, and antigen retrieval was performed in 10 mmol/L Tris-EDTA (pH 9) buffer in a microwave oven for 20 minutes at 100°C. Monoclonal mouse anti-human FXR/NR1H4 antibody (R&D Systems, Oxon UK; clone A9033A; dilution: 1:50) was applied for 1 hour at room temperature.

After washing, immunoreactivity was visualized with Envision kit (Dako B.V., Heverlee, Belgium). The sections were subsequently counterstained with Mayer hematoxylin and evaluated under a light microscopy. As positive control normal human colon was used and as negative controls the primary antibody was omitted.

### Assessement of apoptosis by flow cytometry and caspase 3 activity assay

BE-derived cell cultures CP-18821 (6·10^4 ^cells/well) were plated for 12 hours and incubated in the presence of specific ligands for 48 hours prior to flow cytometric analysis to quantify the percentage of cells which had undergone apoptosis. The following ligands were used: FXR agonist GW4064 (GlaxoSmithKline, Research Triangle Park, USA) at a final concentration of 5 μM, FXR antagonist guggulsterone (Steraloids, Newport, USA) at 20 μM, VDR agonist lithocholic acid (Sigma) at 30 μM and VDR antagonist ZK168281 (Schering, Berlin, Germany) at 1 μM.

Following treatment with ligands, BE cells were labelled with 0.25 μg 7-amino-actinomycin D (7-AAD, BD Biosciences, Switzerland) according to the manufacturer's instructions. Flow cytometry acquisition of events was performed using the CellQuest software with a FACScan equipment (Becton Dickinson, Heidelberg, Germany). In each cell suspension, acquisition was terminated when 10,000 events were analyzed. Debris were discriminated from nonviable cells by a forward scatter (FSC)/FL-3 dot plot in which FL-3 corresponded to 7-AAD-associated fluorescence. In this plot (forward scatter [FSC; *x *axis] versus fluorescence [FL-3; *y *axis]), the combination of size and DNA fluorescence criteria provides a rationale for discrimination between debris and apoptotic cells. Fragments with very low 7-AAD fluorescence were considered to be cell debris with either no or very little DNA and were excluded from cell gates [29].

Cell cultures treated with 2 μM staurosporine (Calbiochem, Luzern, Switzerland) for 24 hours served as positive controls for apoptosis. The control for necrosis was induced in cell cultures with 0.001% sodium dodecyl sulfate for 8 min before the assay (data not shown). Additionally, the 4',6-diamino-2-phenylindole dihydrochloride (DAPI, Sigma) staining was used to confirm the induction of apoptosis: cells were fixed in 4% paraformaldehyde for 20 min, incubated with DAPI 1 μg/mL for 5 min and finally observed with a fluorescence microscope.

To confirm the data on apoptosis issued from the FACS analysis, the activity of caspase 3 was measured in cell cultures exposed to guggulsterone for 8 hours with a fluorometric immunosorbent enzyme assay kit (Roche) according to the manufacturer's instructions.

### Statistics

Data are presented as mean ± SD. All measurements were performed in triplicate. Comparisons between groups were performed using the Student's t-test. A p value ≤ 0.05 was considered statistically significant.

## List of abbreviations

AC: adenocarcionoma; BE: Barrett's esophagus; FXR: farnesoid X receptor; GERD: gastroesophageal reflux disease; VDR: vitamin D receptor

## Competing interests

The author(s) declare that they have no competing interests.

## Authors' contributions

ADG designed the study, carried out parts of the clinical and bench work and prepared the manuscript. JMD, AV, IM, LS and JLF contributed to the clinical part and critically revised the manuscript. FB performed the PCR and flow-cytometry experiments. LRB provided the histopatholocic interpretations and critically revised the manuscript. WNMD performed the immunohistochemistry studies and PSR provided assistance for cell cultures, for the preparation of the study and for the final manuscript. AH contributed to the design of the study, obtained funding for the study and critically revised the manuscript.
